# Natural Polysaccharide-Based Nanodrug Delivery Systems for Treatment of Diabetes

**DOI:** 10.3390/polym14153217

**Published:** 2022-08-08

**Authors:** Aijun Qiu, Yunyun Wang, Genlin Zhang, Hebin Wang

**Affiliations:** 1 College of Chemical Engineering and Technology, Tianshui Normal University, Tianshui 741000, China; 2 Key Laboratory for Green Processing of Chemical Engineering of Xinjiang Bingtuan, School of Chemistry and Chemical Engineering, Shihezi University, Shihezi 832003, China

**Keywords:** natural polysaccharides, diabetes, nanocarriers, drug delivery system

## Abstract

In recent years, natural polysaccharides have been considered as the ideal candidates for novel drug delivery systems because of their good biocompatibility, biodegradation, low immunogenicity, renewable source and easy modification. These natural polymers are widely used in the designing of nanocarriers, which possess wide applications in therapeutics, diagnostics, delivery and protection of bioactive compounds or drugs. A great deal of studies could be focused on developing polysaccharide nanoparticles and promoting their application in various fields, especially in biomedicine. In this review, a variety of polysaccharide-based nanocarriers were introduced, including nanoliposomes, nanoparticles, nanomicelles, nanoemulsions and nanohydrogels, focusing on the latest research progress of these nanocarriers in the treatment of diabetes and the possible strategies for further study of polysaccharide nanocarriers.

## 1. Introduction

Diabetes is a prevalent metabolic syndrome and has become a serious threat to human health [1,2]. It is mainly characterized by hyperglycemia and its complications, often accompanied by the symptoms of glucose, protein, fat, water and electrolyte metabolism disorders [3]. Hyperglycemia is the main marker of diabetes. Long-term hyperglycemia can cause a series of complications such as cardiovascular disease, kidney disease, neurodegeneration and retinopathy [4].

The primary focus of diabetes control is to maintain blood glucose levels within normal range. At present, diabetes is mainly treated with insulin injection and oral hypoglycemic drugs, and commonly used oral drugs mainly include insulin secretagogues, α-glucosidase inhibitors, insulin sensitizers, biguanides, etc. Although repeated and frequent subcutaneous injection of insulin can achieve the purpose of short-term hypoglycemia, it will also lead to some disadvantages, such as local infection, fat removal at the injection site, pressure, insulin resistance, etc., which will make patients feel uncomfortable and have poor compliance [5,6,7]. Similarly, oral hypoglycemic drugs also have many side effects, such as gastrointestinal reactions, liver damage, hypoglycemia, etc. [8]. Therefore, the traditional drug delivery systems still have several limitations [9], and finding effective drug delivery systems with high patient compliance has become a major challenge.

For the past few decades, researchers have been trying to use nanotechnology in diabetes treatment. Various types of nanocarriers, such as liposomes, polymer nanoparticles, micelles, emulsions as well as hydrogels have been developed for delivering hypoglycemic drugs. Nanocarriers have the advantages of low side effects, targeting, long sustained release ability, increased drug permeability and improved efficacy. Compared with synthetic nanocarriers, the natural nanocarriers exhibit higher biocompatibility and biodegradability, greater safety, and better storage and physiological stability. Among natural polymers, natural polysaccharides are widely used because of their diverse functions including biosafety, good tolerance, superior bioavailability and biological activities. More and more evidence indicates that polysaccharide-based nanocarriers have received considerable attention for clinical practice [10,11].

**Figure 1:**
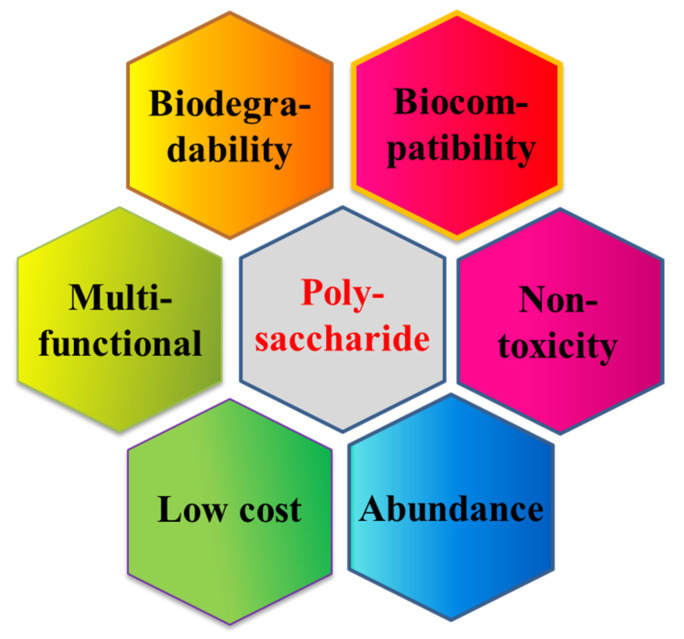
Advantages of natural polysaccharide based nano drug delivery system.

Polysaccharides are natural biopolymers that play different roles in microorganisms, plants and animals [12]. They consist of long monomers of similar type or combinations of other monomer chains [13]. Natural polysaccharides are recognized as safe for targeting delivery due to their superior characteristics, including non-toxicity and non-reactivity, easy availability on a large scale, relative cheapness, significant biocompatibility and extraordinary biodegradability (Figure 1) [14,15]. Due to these excellent properties, natural polysaccharides are widely used in the designing of nanocarriers and have a wide range of applications in therapeutics, diagnostics, delivery and protection of bioactive compounds or drugs [16]. In addition, some natural polysaccharides can provide targeting mechanisms due to site specific enzymatic degradation, receptor interaction and binding, environmental trigger, mucosal adhesion and transport, etc. [17,18].

Over the past few decades, the interest of the pharmaceutical therapy utilizing nanotechnology in diabetes treatment has been dramatically increased. Different types of hypoglycemic nanocarriers have been developed, and natural polysaccharide nanocarriers have attracted wide attention because of their higher biocompatibility and biodegradability, greater safety, better storage and physiological stability [19]. In this review, a variety of polysaccharide-based nanocarriers were introduced, including nanoliposomes, nanoparticles, nanomicelles, nanoemulsions and nanohydrogels, focusing on the latest research progress of these nanocarriers in the treatment of diabetes and the possible strategies for further study of polysaccharide nanocarriers.

## 2. Classification of Polysaccharides

Polysaccharides are composed of many similar types or different types of monosaccharides, through different glycosidic bond binding sites and configurations of monosaccharides, which leads to the extremely rich and complex structure of polysaccharides. According to the monosaccharide composition of polysaccharides, they can be divided into two types. Polysaccharides consisting of a single type of monosaccharide are defined as homopolysaccharides, while polysaccharides consisting of more than one type of monosaccharide are heteropolysaccharides [20]. For example, glycogen is an important homopolysaccharide existing in the human body, which has the ability to store glycose. Its repeating unit is only glucose, and glycose units were conjugated via α-1,4-glycoside bonds as chain structure and via α-1,6-glycoside bonds as branches [21]. Glucan is also a homopolysaccharide composed of repeating glucose units linked by the glucosidic bonds. Starch is a homopolysaccharide that exists in plants for storing glycose. It has different branching distribution and molecular weight compared with glycogen [22]. Another common plant homopolysaccharide is cellulose, which is linked by β-1,4-glycosidic bonds as a chain structure. Dextran is a bacterial homopolysaccharide with glycose units conjugated via α-1,6-glycoside bonds, and branch chains are linked by α-1,3-glycosidic bonds [20]. In contrast, heteropolysaccharides can be hydrolyzed into at least two different monosaccharides. For example, chitosan is a natural heteropolysaccharide derived from deacetylation of chitin. It is comprised of D-glucosamine and N-acetyl-D-glucosamine units connected with β-(1–4) glycosidic linkages [23]. Pectin is a heteropolysaccharide abundant in the cell wall of plants, composed of (1,4)-linked α-D-galacturonic acid residues and variety of neutral sugars such as rhamnose, galactose and arabinose [24]. Sodium alginates consist of α-L-guluronic acid (G) and β-d-mannuronic acid (M) [20]. Compared with homopolysaccharides, heteropolysaccharides have various combinations of different monosaccharides, which provide opportunities to regulate their structure and obtain suitable chemical structures that meet the requirements of physical and chemical properties [25,26]. Several natural polysaccharides commonly used in the preparation of nanocarriers are introduced below.

### 2.1. Chitosan

**Figure 2:**
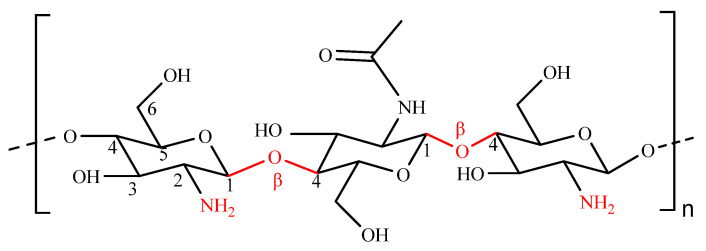
The structure of chitosan.

Chitosan (CS) is generally recognized as safe (GRAS) natural polysaccharide by the US Food and Drug Administration (FDA) with great application potential and advantages, such as abundant sources, biocompatibility, biodegradability, non-toxicity, low cost, and good mucoadhesion [27,28,29]. Therefore, chitosan and its derivatives have been widely used for food, medicine and other fields, especially for nanodrug delivery systems [30,31]. CS is a cationic linear heteropolysaccharide comprised of D-glucosamine and N-acetyl-D-glucosamine units connected with β-(1–4) glycosidic linkages (Figure 2). The amino functional groups in CS play an important role in the biochemical regulation and electrostatic interactions in nanodrug delivery systems and provide solubility in slightly acidic solutions [32]. Even though it has a positively charged amino group, CS holds very little immunogenicity activity; as a result, it is being used as a bioadhesive material that adheres to mucosal cell, enhances the withholding characteristics and finally penetrates into the interior of cell. The adhesive property of CS provides an excellent platform for drug delivery via enhancing nasal and intestinal absorption [33]. Mucoadhesion of CS is accomplished mainly by electrostatic interaction followed by hydrogen bonding and hydrophobic interaction. Moreover, the presence of hydroxyl and primary amine groups on the backbone of CS also provides ample opportunities for various chemical modifications [34]. CS‘s’ positive charges also empower it to expand the opening of epithelial tightfitting intersections and broaden paracellular passageways corridors, supporting the transportation of drugs into the targeting tissues [35]. Above all, CS has become one of the most widely used as a carrier material for fabrication of nanocarriers at present.

### 2.2. Glucan

**Figure 3:**
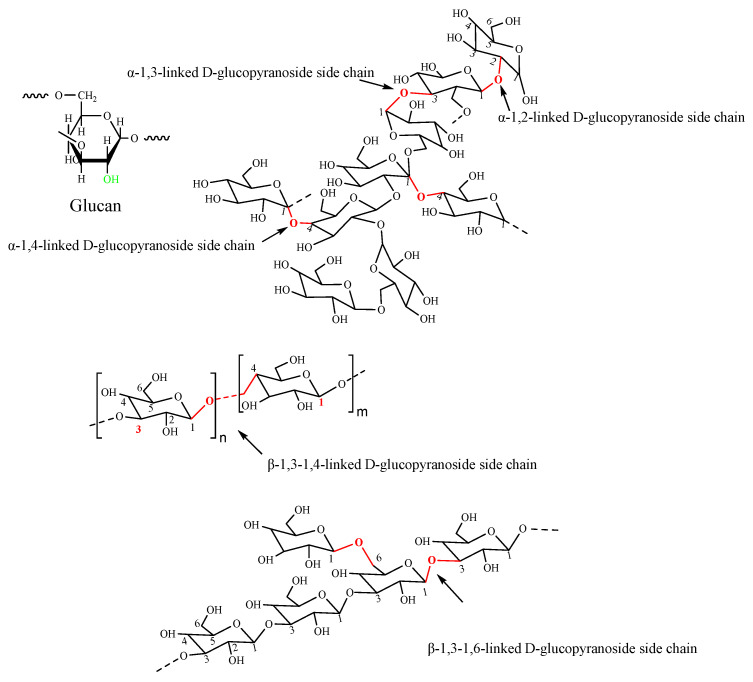
The structures of α-D-glucan and β-D-glucan.

Glucan is a homopolysaccharide of glucose units linked by the glycoside bond. According to the type of monosaccharide residues anomeric structure, glucan can be divided into α-D-glucan, β-D-glucan, and mixed α,β-D-glucan (Figure 3) [36]. The complexity of glucan depends on structural differences in the chain conformation, branching degree, molecular weight and functional groups. All these differences result in glucans with different properties [37]. Dextran is a generic term for a family of glucans, consisting of α-D-(1–6) glycosidic linkages between glucose monomers with branches from α-1, 2, α-1, 3, and α-1, 4 linkages [38,39], which is the most used and the first commercialized microbial polysaccharide. It is known as a physiologically harmless, excellently soluble, superior biodegradable, high biocompatible and non-immunogenicity polysaccharide with abundant hydroxyl groups that facilitate a wide range of modifications.

Dextran nanoccarriers can promote drugs dissolution and penetration through the gastrointestinal (GI) membrane. Unlike other polysaccharides, dextran is almost impossible to break down by the common amylase such as salivary amylase or malt amylase. It can be only depolymerized by dextranase in the lumen of the large intestine, liver, spleen, and kidney [40]. Thus, dextran-based drug delivery systems can protect drug molecules from chemical and enzymatic degradation in the stomach and small intestine, increasing uptake by the intestinal epithelium and improving oral bioavailability [41].

### 2.3. Pectin

**Figure 4:**
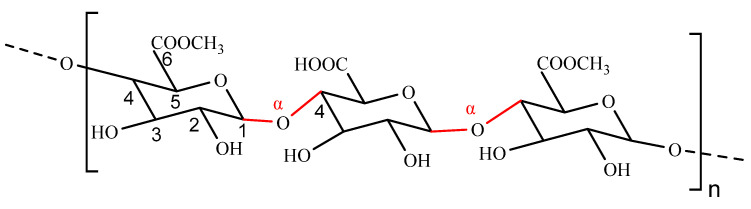
The structure of pectin.

Pectin is a hydrophilic anionic and linear natural polysaccharides, which is composed of D-galacturonic acid liked with α-(1–4) glycosidic bonds (Figure 4) [42]. The degree of methylation (DM) is a crucial parameter that affects the properties of pectin. They are usually classified into two main classes of high methoxyl pectin (HMP, more than 50% of the carboxyl groups are methylated, DM > 50%) and low methoxyl pectin (LMP, less than that degree of methylation, DM < 50%) on the basis of DM as well as the number of methoxyl groups [43]. Pectin is considered to be a promising carrier in the nanoencapsulation process. It can protect drugs from enzymatic proteolysis by presenting protease inhibiting action and increase the permeation capacity of nanoparticles, crossing the upper gastrointestinal intact. As an colon-specific drug delivery, it is degraded and absorbed in intestine by pectinolytic enzymes formed via colonic microflora [17,44]. Due to these characteristics, pectin as an important material for the development of colon-specific drug delivery system has attracted the attention of researchers [45,46].

### 2.4. Alginate

**Figure 5:**
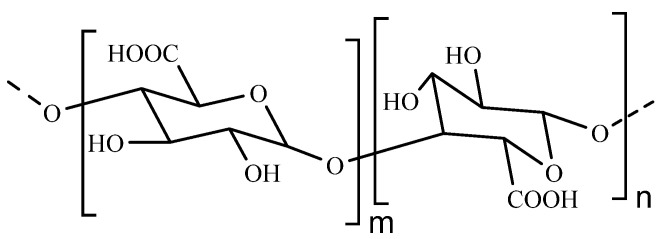
The structure of alginate.

Alginate (ALG) is a linear anionic natural polysaccharide extracted from brown seaweed, consisting of irregular ratios of (1–4)-α-L-guluronic acid units (G) and (1–4)-β-D-mannuronic acid units (M) linked by glycosidic bonds (Figure 5) [47,48]. This gives alginate different properties from other natural polymers, as the different proportions and distributions of these three types of blocks (homopolymeric MM and GG, and heteropolymeric MG blocks) determine the chemical and physical properties of alginate molecules [49].

ALG is able to produce gels in contact with bi- and trivalent cations (e.g., Al^3+^, Ba^2+^, Sr^2+^ or Ca^2+^), by cross-linking the carboxylate groups of the guluronate groups on the polymer backbone [50,51]. Interestingly, this polysaccharide can also form gels by hydrogen bonding in acidic media. ALG is characterized by good biodegradability, biocompatibility, low immunogenicity, good adhesion and non-toxicity, and has attracted extensive attention in preparing drug delivery vehicles, and especially oral insulin nanoparticles. They can be easily reshaped into many flexible biomaterial forms such as particles (nano, micro, bead), fibers, films, sponges, injectable gels, etc. [49]. Various alginate-based nanoparticles have been prepared by ionic gelation or polyelectrolyte complexation. In recent years, ALG has been widely used in nanomedicine delivery systems, either alone or blended with other polysaccharides, including chitosan, pectin and carrageenan, to combat a variety of diseases [17,46]. 

### 2.5. Hyaluronic Acid

**Figure 6:**
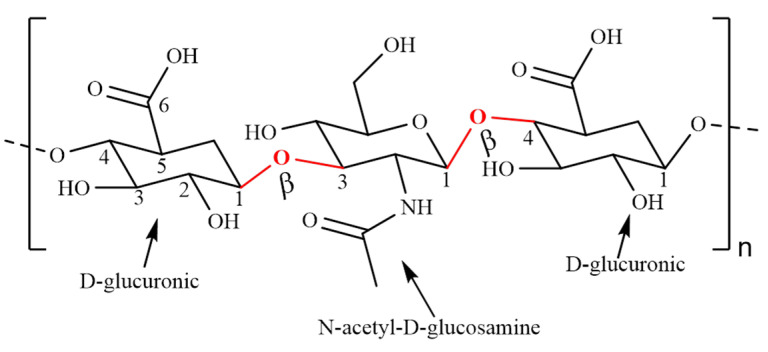
Chemical structure of hyaluronic acid.

Hyaluronic acid (HA) is a linear, negatively charged polysaccharide composed of alternately linked D-glucuronic and N-acetyl-D-glucosamine repeating units via β-1,3 and β-1,4 glycosidic bonds (Figure 6) [52]. HA is the simplest glycosaminoglycan (GAG) present in nature. Even though HA has the simple linear chain, it shows several important biomechanical properties playing key roles in cell motility, proliferation and regulates cell–cell adhesion [53,54]. HA is found throughout the body, especially in the skin, vitreous of the eye and synovial fluid. It is an essential structural molecule of the human body, which contains more than 15 g in total [55]. HA can function as a lubricant and shock absorber, therefore it is approved by the FDA as an injectable to treat knee pain in osteoarthritis or for facial implants to make skin appear smoother. It is necessary for embryonic development, wound healing repair, and inflammation [56,57]. HA has attracted significant interest in development of drug delivery systems because of its intrinsic physicochemical and biological properties [58].

### 2.6. Starch

**Figure 7:**
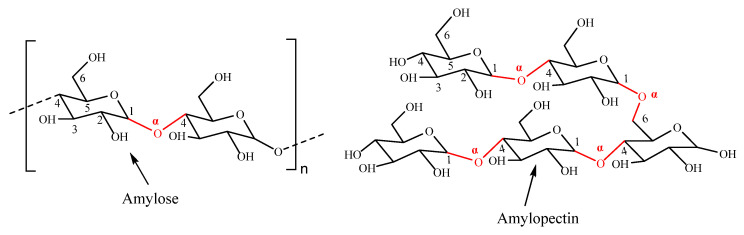
Structure of amylose and amylopectin.

Starch is one of the most available natural polysaccharides. It has excellent biocompatibility, biodegradability, non-toxicity and low cost. Due to its gel-forming and film-forming properties, starch has become one of the most studied polysaccharides. The structure of starch consists of two different monomers, namely amylose and amylopectin. Amylose is a linear polymer with a-(1,4)-linked D-glucose units while amylopectin is a highly branched polymer composed of a-(1,4)-linked D-glucose units branched by a-(1,6) glycosidic bonds (Figure 7). The physicochemical and functional properties of starch depend on the proportion of these polysaccharides in starch [17,59].

Generally, native starch has attracted extensive attention in drug delivery systems (DDS) because of their unique advantages. However, native starch has suffered from some disadvantages such as the fast release due to its hydrophilic nature, substantial swelling and rapid enzymatic degradation in biological systems, and thus is considered unsuitable in some controlled release systems. These disadvantages could be overcome through modification of native starch. Several modified starches in the market, such as dextrin, maltodextrin and octenyl succinic anhydride (OSA) modified starches, can be used for drug nanodelivery systems (DNDSs) [60,61,62]. Due to their carefully designed structure, modified starches have been used to fabricate different nanocarriers that carry drugs to treat diseases.

### 2.7. Cellulose

**Figure 8:**
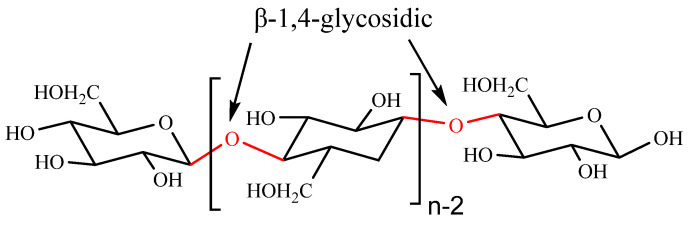
Structure of cellulose.

Cellulose is the most abundant natural polysaccharide on Earth, and is extensively obtained from the primary cell walls of many algae, bacteria, plants, and fungi [63]. Cellulose is a linear polysaccharide consisting of β-1,4-glycosidic linked D-glucose units (anhydroglucose unit) (Figure 8). It is widely documented as a high biocompatible, superior biodegradable, less toxic, and GRAS with remarkable physical, mechanical and chemical characteristics in the medical field, which makes it an appropriate candidate for the successful entrapment and transport of drugs. However, the poor solubility of natural cellulose in water is a major constraint limiting its further application. For overcoming this problem, different modified celluloses are prepared through physical, chemical or biochemical methods [64]. A variety of modified celluloses such as cellulose nanocrystals (CNCs), nanofibrillar cellulose (NFC) and carboxymethyl cellulose (CMC) can be used for drug nanoencapsulation and their delivery to different disease targets [17].

Nanocellulose is one of the most promising green materials to be derived from abundant and inexhaustible cellulose [65,66]. In recent years, nanocellulose has been designed into multi-dimensional structures, including one-dimensional (nanofibers, microparticles), two-dimensional (films), and three-dimensional (hydrogels, aerogels) materials for drug delivery, wound healing, and tissue engineering [67]. Nanocellulose-based materials have attracted extensive attention due to their adaptable surface chemistry, high surface area, biocompatibility, and biodegradability [67,68].

## 3. Types of Nanocarriers Used for the Treatment of Diabetes

Nanoencapsulation systems have shown surprising advantages and have been widely used in different fields. They can encapsulate both hydrophobic and hydrophilic compounds, and improve stability, reduce toxicity and protect them from degradation. The drugs can either be encased in the nanocarriers matrix or attached onto their surface, and will be released for the target site. Nanocarriers show so many advantages for drug delivery, including appropriate particle size, tunable surface charge, large surface area and drug loading capacity. These properties make nanodrug delivery systems ideal candidates for detection, diagnosis and treatment of disease [17,69,70,71].

**Figure 9:**
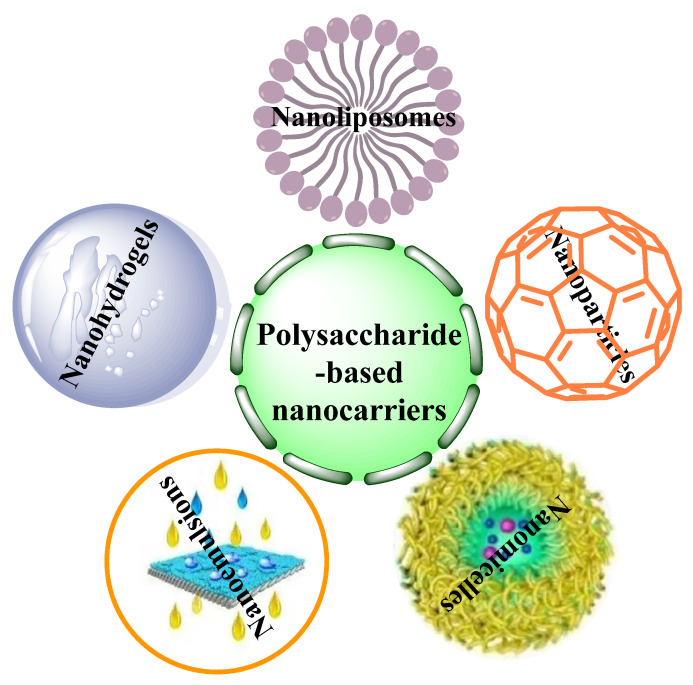
Polysaccharide-based nanocarriers.

In addition to the above-mentioned, polysaccharide-based nanocarriers also exhibit succeeding advantages such as less toxicity, safety, higher emulsifying capacity, improved encapsulation efficiency, targeted drug delivery and controlled release, and accelerated intestinal absorption [72]. Polysaccharide-based nanocarriers are mainly divided into nanoliposomes, nanoparticles, nanomicelles, nanoemulsions and nanohydrogels (Figure 9).

### 3.1. Polysaccharide-Based Nanoliposomes

Liposomes are self-assembled bilayer vesicles that have attracted great interest as potential carriers for many therapeutic drugs. Due to their low permeability, drug molecules can be encapsulated either in the hydrophilic core or the hydrophobic lipid bilayer, or bind to the surface of the vesicle [73]. In the late 1970s, liposome-coated insulin was administered by the oral route to diabetic animals. Results showed that a certain amount of insulin could reach the circulation [74]. As a carrier, liposomes have many advantages of non-toxicity, non-immunogenicity and biodegradation, and can encapsulate hydrophilic and hydrophobic drugs. Moreover, liposome surface can be modified to increase the specificity of drug delivery. However, nanoliposomes have low stability and easy loss of encapsulated drugs, which limits their application. These disadvantages can be overcome by coating biopolymers and bioadhesive materials. Polysaccharide-based nanoliposomes are used as nanocarriers to enhance drug stability and targeted delivery due to their high charge density and adhesion properties. Nanoliposome outer layers coating polysaccharides can avoid the degradation of liposome membrane in GIT (gastrointestinal tract) surroundings, because polysaccharides can retard the fluidity of nanoliposome membranes, thus improving their stability. The electrostatic networks between polysaccharides and phospholipids are able to hinder the permeability of the nanoliposome membrane, reducing drug leakage [17,75].

As an example, Prof Subbu Venkatraman’s group [76] developed a multi-layered insulin loading strategy on an anionic nanoliposome surface based on electrostatic interaction with chitosan. Chitosan was used for the alternating cationic layers to hold the insulin (negative charge at the pH employed), the layer-by-layer (LBL) coated nanoliposomes insulin loading rate was 10.7% and offered superior protection with limited release in simulated gastric fluid (SGF) (only 6% in 1 h), and simulated intestinal fluid (SIF) (2% in two weeks). The LBL coated liposome had excellent stability for 4 weeks in PBS pH 7.4 at 37 °C. The outermost chitosan layer of the layer-by-layer coated liposome facilitated cellular uptake and transport by Caco-2 cells, and the transported insulin demonstrated retention of bioactivity through glucose uptake assay performed on 3T3 L1-MBX adipocytes. These layer-by-layer coated liposomes could protected insulin during intestinal penetration and ensure its insulin payload reached the systemic blood circulation, thus indicating the potential application of these nanoparticles in the field of oral protein delivery. In a research work, Maestrelli et al. [77] developed two different metformin-loaded chitosomal and niosomal, but they did not have a sustained release function. Then, the two colloidal dispersions were coated with calcium alginate beads, which significantly reduced drug release at gastric level (from 18 to up to 30%) and sustained release in simulated intestinal fluid, which was adjusted by changing the proportion of calcium alginate in the beads. Studies in rats showed that alginate beads coated chitosomal and niosomal significantly improved the hypoglycemic effect of metformin. The drug in-niosomes-alginate bead formulation was more capable of maintaining glucose levels over time, allowing for reduction of its dose and associated side effects as well as improving patient compliance. In another investigation, Cosco et al. [78] designed a hyaluronic acid-based nanoliposome (HA-DPPE), which was obtained by connecting hyaluronic acid and phospholipid DPPE, and then the DPPE moieties of HA-DPPE were inserted into DPPC-Chol’s liposomal bilayer to form HA-coated nanoliposomes. These HA-coated nanoliposomes improved stability in serum compared to that of plain liposomes. This work describes an innovative strategy for coating vesicular systems to confer them simultaneously with long circulation properties and selective targeting towards HA-receptors.

### 3.2. Polysaccharide-Based Nanoparticles

There are general agreements that nanoparticles (NPs) are solid colloidal particles composed of macromolecules ranging in size from 10 nm to 1000 nm [79]. However, nanomedicine generally refers to devices < 200 nm, the width of microcapillaries. NPs are used in a broad spectrum of applications. They are contained in many products and used in various technologies. NPs have significant advantages to carry drugs and other cargo in the body. Typically, the drug of interest is dissolved, encapsulated, adsorbed, attached and/or coated on a nanomatrix. In recent years, many researchers have successfully fabricated polysaccharide-based nanoparticles encapsulating drugs for the treatment of diabetes.

For example, Lopes et al. [80] designed and prepared an insulin-loaded alginate/dextran sulfate (ADS) -NPs, and double-coated it with chitosan (CS) and albumin (ALB). The nanosystem was characterized by pH sensitivity and mucus adhesion, which enabled us to prevent 70% of in vitro insulin release under simulated gastric conditions and allowed sustained insulin release in simulated intestinal conditions. The pH value and time-dependent morphology of the NPs was correlated to the insulin release and permeation. Dual CS/ALB coating of the ADS-NPs showed enhanced interaction with intestinal cells, resulting in a higher insulin permeability across Caco-2/HT29-MTX/Raji B cell monolayers. The developed nanosystem showed clinical potential for the oral delivery of insulin and therapy of type 1 diabetes mellitus. The polysaccharide-based nanoparticles have good clinical potential for oral delivery of insulin for treatment diabetes. In another example, a core-shell-corona nanoparticle of alginate and succinyl chitosan was developed through encapsulating quercetin into nanoparticles with ionic crosslinking [81]. Overall, 95% of quercetin was effectively encapsulated in this nanoparticle. The release of quercetin was significantly pH sensitive, with an average 16–27% release of within 2 h at gastric pH 1.2, whereas a significant release of 88–95% at pH 6.8 and 7.4 of simulated intestinal fluid. Compared with free oral quercetin, peroral delivery of these quercetin nanoparticles has a significant hypoglycemic effect and effective maintenance of glucose homeostasis in diabetic rats. This suggests that the nanoparticles could be an effective oral quercetin carrier for the treatment of diabetes.

Bhattacharyya et al. [82] designed an efficient oral insulin delivery carrier based on polyurethane-alginate/chitosan (PU-ALG/CS). The insulin-loaded PU-ALG/CS nanoparticles had an encapsulation rate of more than 90%, effective control of insulin release, maintained hypoglycemia in diabetic mice, and improved insulin bioavailability. PU-ALG/CS nanoparticles were also found to be safe according to the acute toxicity studies. Akhlesh Kumar Jain et al. [83] designed and developed a series of starch-based nanoparticles for the intranasal delivery of insulin. The NPs can rapidly release insulin and exert hypoglycemic effects quickly and continuously until 6 h. This rapid release is useful for drugs of short in vivo half-lives, and provides therapeutic concentrations for a long time, thus achieving a better therapeutic effect. The degree of cross-linking can modulate insulin release from these carriers and affect in vivo performance. The release rate and the higher associated surface area might act synergistically on efficient insulin delivery, and when combined with permeation enhancers, the release rate of starch NPs can be greatly amplified, to make starch NPs a highly efficient trans-nasal carrier for insulin. Santhosh Kumar Chinnaiyan et al. [84] prepared metformin-loaded pectin nanoparticles (PCMNPs) for sustained treatment of type 2 diabetes mellitus (T2DM). The encapsulation rate of PCMNPs was 68 ± 4.2%, which showed favorable sustained release characteristics in vitro. In addition, the nanoparticles were fairly stable in the presence of excess bovine serum albumin, suggesting that nanoparticles may also be stable in the bloodstream. The hemolysis rates induced by metformin and placebo PCNPs were less than 5%, indicated that PCMNPs are hemocompatible and safe orally. Therefore, the designed nanoparticle system may have advantages in terms of prolonged release, reducing dose frequency and improving patient compliance. Zhe Zhang et al. [85] developed a novel nanoparticle for oral- and liver-targeted delivery of insulin. The nanoparticles were prepared from cholic acid, quaternary ammonium modified chitosan derivatives and hydroxypropyl methyl cellulose phthalate (HPMCP). The insulin loading rate and loading capacity of nanoparticles were 90.9% and 18.2%, respectively, and the diameter was 239 nm. Cell culture studies showed that the cholic acid group effectively enhanced the transport of nanoparticles through Caco-2 monolayer, and greatly increased the absorption of nanoparticles in HepG-2 cells through the bile acid transporter mechanism. In this system, chitosan derivatives have adhesive properties and can enhance the absorption of loaded insulin in the gastrointestinal tract. HPMCP can protect insulin from degradation and denaturation in the harsh environment of the stomach. The treatment of diabetic mice displayed that the hypoglycemic effect of the oral nanoparticle group could be maintained for more than 24 h, and its pharmacological effectiveness was about 30% compared with that of the insulin injection group.

### 3.3. Polysaccharide-Based Nanomicelles

In recent years, polymeric micelles self-assembled from amphiphilic polymers have been widely applied in drug delivery fields due to their improved pharmacokinetics and biodistributions. The core of micelles can accommodate hydrophobic drugs, and the shell is a hydrophilic brush-like corona that makes the micelle water soluble, thereby allowing delivery of the poorly soluble contents [69]. Therefore, polymeric micelles provide a feasible platform to prevent protein degradation by various enzymes. Polymeric micelles are used medically as carriers for drugs because they provide solubility and thus better intestinal permeability of micelles. Nanomicelles are typically spherical, but can sometimes take other shapes, such as cylinders and ellipsoids. The small size and shape of nanomicelle is only possible due to the molecular geometry of the particle. The shapes formed also depend on the ionic strength, surfactant concentration, and pH strength of the solutions they are placed in. Some interesting designs have been reported using natural polysaccharide micelles, for example, Na Wen et al. [86] designed and synthesized a glucose responsive micelle–hydrogel system for the synergistic treatment for diabetes and vascular diabetic complications. Zwitterionic dialdehyde starch-based micelles (SB-das-VPBA) were synthesized by single electron transfer-living radical polymerization (SET-LRP). The hydrophilic segment sulfobetaine (SB) and hydrophobic segment 4-vinyl phenylboric acid (VPBA) were grafted to the skeleton of dialdehyde starch (DAS). Then, chitosan/dialdehyde starch derivative (CS/SB-DAS-VPBA) micelle–hydrogel was synthesized by Schiff-base bond. The micellar–hydrogel system was developed by loading insulin and nattokinase. The synergistic treatment system could provide continuous insulin release and rapid nattokinase release in 3 g/L glucose solution, greatly improving the efficiency of diabetes and vascular diabetes complications. In vitro administration and blood clots dissolution behaviors were measured. The results showed that the micellar and hydrogel synergetic therapy system not only had the characteristics of glucose responsive insulin delivery, but also provided good thrombolytic capacity. Therefore, this micelle–hydrogel synergistic therapy system can be used as a platform for the treatment of diabetes and vascular complications of diabetes. In another research, Sabyasachi Maiti et al. [87] synthesized and characterized the xanthan gum copolymer with C16 alkyl linked branches. The copolymer was self-assembled into spherical nanomicelles in water and incorporated almost 100% glibenclamide in the lipophilic core, Xanthan gum was used as a hydrophilic shell-forming material. Copolymers could enhance the solubility of drugs and prolonged the release time of drugs in vivo pH conditions. Pharmacodynamics evaluation showed that the formulations had great potential in long-term control of blood glucose level in animal models. In summary, glyburide is an antidiabetic drug with poor water solubility, and its copolymer micelles entrapment into hydrogel particles were a promising approach for the control and effective treatment of diabetes.

Muhammad Usman Akbar et al. [88] prepared curcumin-loaded mixed polymeric micelles based on chitosan, alginate, maltodextrin, Pluronic F127, P123 and Tween 80 by thin-film hydration method to improve the solubility, chemical stability and bioavailability of curcumin. This micelle reduces elevated blood glucose level and lipid profile, maintained rat body weight, high density lipoprotein cholesterol levels, various biochemical parameters and accelerated the process of wound healing. The curcumin-loaded mixed micelles have a favorable inhibitory effect on histopathological changes in liver, kidney, and pancreas. Compared to free pure curcumin, the newly developed curcumin-based mixed micelles proved to have superior therapeutic potential and excellent healing efficacy.

### 3.4. Polysaccharide-Based Nanoemulsions

Nanoemulsions are very versatile systems for encapsulating drugs dissolved in the disperse phase [89]. They can enhance the therapeutic efficacy of the drug and reduce the adverse effect and toxic reactions [90]. Nanoemulsions are dynamically stable emulsions with droplet sizes in the nanometer scale, consisting of two or more insoluble phases, such as oil, water, and emulsifier (polysaccharide) [91]. The purpose of using polysaccharides as emulsifiers is to minimize the interfacial tension among the aqueous phases and the lipid phase and ultimately reduces the droplet size [92]. They can maintain the long-term stability of the nanoemulsion by means of steric hindrance and electrostatic force. Therefore, polysaccharide-based nanoemulsions are recognized as ideal carriers for functional compounds and drugs [93]. They have the characteristics of dynamic stability, large specific surface area, transparency and are easy to prepare drug nanodelivery systems for therapeutic drugs [17,94].

Sandeep Kumar et al. [95] prepared a water-in-oil-in-water metformin nanoemulsion (metformin-loaded alginate nanoemulsion) by an emulsion cross-linking technology, which promoted the gastrointestinal absorption and intestinal permeability of drugs and improved the therapeutic effect of diabetes. Metformin loading and encapsulation efficiency of nanoemulsions were 3.12% and 78%, respectively. In vitro drug release studies and in vivo efficacy tests showed that metformin-loaded alginate nanoemulsions had better efficiency and reaction than free pure metformin. The efficacy of nanoemulsions were about three times higher than that of free pure metformin. In this study, alginate was gelated in aqueous solution, and the bioavailability of metformin was increased through encapsulation. The nanoformulations had the characteristics of simple preparation, economy, biocompatibility, biodegradability and high encapsulation efficiency. Xiaoyang Li et al. [96] devised alginate/chitosan-coated nanoemulsions for oral insulin delivery. The nanoemulsion was prepared using Labrafac^®^ CC, phospholipid, Span™ 80 and Cremorphor^®^ EL by water-oil-water (W/O/W) homogenization method. The nanoemulsion was coated by adding calcium chloride and chitosan to the nanoemulsion dispersion that contained alginate. The particle size of the coated nanoemulsion was about 488 nm and the insulin encapsulation rate was 47.3%. In vitro leakage study showed that the nanoemulsion was well preserved in simulated gastric juice. Moreover, the hypoglycemic effect of oral coated nanoemulsion was significantly prolonged compared with subcutaneous insulin. Alginate/chitosan coating on nanoemulsions could protect the stability of insulin and improve permeability and bioadhesion in gastrointestinal. The results suggested that alginate/chitosan-coated nanoemulsion was a promising oral delivery system for peptides and proteins.

### 3.5. Polysaccharide-Based Nanohydrogels

Nanohydrogels are cross-linked three dimensional structure composed of nanosized polymeric networks [97]. Hydrogels have high water content and soft networks, which can minimize the tissue irritation or cell adherence. Due to their porous structure and high water content, hydrogels can be encapsulated water-soluble active substances such as proteins, peptides, siRNA, DNA and vaccines into their 3D networks [98]. They have been widely applied in drug delivery fields due to their higher mechanical stability, excellent drug loading and stimulating controlled-release capability, which make them more suitable choice for their many application fields. Polysaccharide-based nanohydrogels have attracted wide attention as drug delivery systems due to their biodegradability, biocompatibility, availability, low cost, non-toxicity, efficient drug release, and similarity with extracellular matrix macromolecules [17,99]. Polysaccharides can easily form nanohydrogels by chemical or physical crosslinking (including hydrogen bonding and ionic interactions) or a combination of both, capable of imbibing large amounts of water or biological fluids. In addition, different polysaccharides are composed of several functional groups that can be used for bioconjugation to produce novel nanohydrogels [100].

As an example, Hui-Peng Lim et al. [101] prepared pH responsive composite hydrogel beads based on natural polysaccharides (alginate and κ-carrageenan) for insulin aspart delivery. At pH 1.2, the composite hydrogel beads successfully retained insulin aspart through electrostatic interaction between positively charged insulin aspart and the negatively charged sulfate group of the κ-carrageenan polymer. At pH 7.4, insulin aspart was released in a gradual manner. With the increase of κ-carrageenan concentration during the formation of hydrogel beads, the release curve approached zero-order dynamics. About 65% of the insulin aspart remained bioactive after the composite hydrogel beads were incubated in an acidic simulated gastric medium. The results suggest that alginate /κ-carrageenan composite hydrogel beads were a promising oral insulin delivery system. Kunhua Lin et al. [102] designed and synthesized a hydrogel based on pullulan (a linear glucan) for insulin delivery. Firstly, carboxylated pullulan (CPUL) was obtained by carboxylating pullulan with succinic anhydride. A glucose-sensitive covalent modified CPUL and concanavalin A (ConA) hydrogel was prepared by amidation between the COOH group of CPUL and the NH_2_ group of ConA with a safe and mild 1-ethyl-3-(3-dimethyl aminopropyl) carbodiimide hydrochloride/N-hydroxysuccinimide (EDC/NHS) activation method. Insulin-loaded CPUL-ConA hydrogel was prepared by absorption of insulin in situ. In vitro release analysis confirmed the intelligent control of insulin release from insulin/CPUL-ConA hydrogels due to specific ConA-glucose binding. Insulin/CPUL-ConA hydrogels showed the characteristic viscoelastic and shear thinning properties of covalently modified hydrogels. Scanning electron microscopy showed that the hydrogel had a cross-linked network structures containing uniform pores and insulin particles. This work provides a pullulan-based glucose-sensitive hydrogel that intelligently controls insulin release in response to changes in glucose concentration. The hydrogels could be used in a closed-loop system for diabetes therapy.

Yu Yang et al. [103] prepared and explored an oral insulin administered polysaccharide (carboxymethyl β-cyclodextrin-g-carboxymethyl chitosan) hydrogel for the treatment of type 2 diabetes mellitus (T2DM). The results showed that long-term oral administration (four weeks) of insulin polysaccharide hydrogel could significantly alleviate the symptoms of polyphagia, polydipsia, polyuria and weight loss in diabetic mice. It can significantly reduce fasting glucose level, ameliorate insulin resistance and improve insulin sensitivity of T2DM mice. This insulin polysaccharide hydrogel can also regulate lipid metabolism and prevent diabetes nephropathy complications. In addition, insulin polysaccharide hydrogel could significantly improve the antioxidant capacity of diabetic mice, and reverse the histological deterioration of kidney and pancreas in diabetic mice. These results suggested that insulin polysaccharides hydrogels may have good anti-diabetic activity and may be a candidate drug for the treatment of T2DM. Xiaoliang Qi et al. [104] proposed an improved food gum (Salcan) based hydrogel for oral insulin delivery. During hydrogel formation, the dosage of salcan can control the modulus, morphology and swelling properties of hydrogel. Insulin was introduced into the hydrogel using swelling diffusion, and in vitro insulin release showed that insulin release was inhibited under acidic conditions, but significantly increased at neutral pH. Cell viability and toxicity tests demonstrated that salcan hydrogel was biocompatible. Oral administration of insulin-loaded salcan hydrogel resulted in a sustained decrease in fasting blood glucose levels in diabetic rats 6 h after administration. These results contribute to further development of salcan-based hydrogels as oral insulin delivery vectors.

In addition, polysaccharide-based nanohydrogels also have many applications in wound healing under diabetic conditions. For example, Yogendra S. Padwad et al. [105] studied and developed a bamboo cellulose nanocrystal-based nanobiocomposite hydrogel (NBCH), which can be used as an effective material for healing wounds in streptozotocin induced diabetic mice model. The developed NBCH accelerated diabetic wound healing within 18 days compared to various control groups requiring longer healing time, regulated the expression of certain pro-inflammatory cytokines and growth factors that delay diabetic wound healing, reduced inflammatory response, and promoted early hyperplasia, collagen formation and epithelialization. The results indicated that the NBCH have great potential as an ideal wound dressing for efficient and rapid healing in patients with diabetes. For another example, Adrian T. Press et al. [106] used bacterial nanocelluloses (BNC) as wound dressing to load α -13′-COOH (α-Tocopherol-Derived Metabolite), which was directly applied to wounds using a splinted wound mouse model under the condition of diabetes, to investigate their effects on wounds’ proinflammatory microenvironment and wound healing. The results showed that BNC could control the release of α -13′-COOH and promote wound healing and wound closure. These results indicate that α -13′-COOH combined with BNC as a potentially wound dressing for the advanced treatment of skin injuries.

In summary, natural polysaccharides play a very important role in the nanodrug carriers and achieve good results in the treatment of diabetes. More relevant studies have been summarized in Table 1.

## 4. Conclusions and Perspective

Diabetes is a chronic metabolic disease, with 537 million people living with it in 2021, according to the International Diabetes Association. It is predicted that by 2045, 700 million people will have the disease worldwide. Diabetes has a devastating impact on individuals, societies, countries or territories, resulting in more than 4 million deaths each year. It affects all ages, communities and continents. Countries or territories must urgently take effective actions to improve the prevention and management of diabetes [153]. Diabetes requires lifelong treatment, but long-term use of hypoglycemic drugs will cause damage to liver, kidney, intestines and other diseases, as well as hypoglycemia. It is of great significance to develop nano drugs with low toxicity for the treatment of diabetes. In recent years, natural polysaccharides (NPLS) have been regarded as ideal candidates for hypoglycemic drug delivery system owing to their GRAS materials, excellent biocompatibility, non-toxicity, low cost, and improved solubility and storage capacity.

Polysaccharides are the most abundant natural biopolymers in the biosphere, from a wide range of sources including plants, animals, microorganisms, marine organisms, etc. [154]. Polysaccharides have enormously large and broad applications in food and medicine and other fields, and their use depends on the unique physicochemical properties, and lower costs than synthetic polymers. Therefore, polysaccharides have great potential for exploitation and utilization [155].

NPLS, including pectin, starch, alginate and chitosan, are mainly recognized as excellent bioadhesive matrices. Drug-loaded nanocarriers prepared through bioadhesive polysaccharides could enhance the resistance time as well as cellular absorbance/uptake of the drugs [17]. Chitosan has a unique property of adhering to mucosal surfaces and penetrating to tight junctions between endothelial cells. HA has targeting ability to specific cells by binding with cell surface receptors and can be utilized for targeted drug delivery. Additionally, pectin has unique features for drug delivery, such as muco-adhesiveness, ease of dissolution in basic environments, the ability to form gels in acidic environments, etc. [156].

Moreover, a development direction of polysaccharide NPs is the combination with polymer materials. When a polymer is connected to a polysaccharide NPs to form a polysaccharide/polymer NP system, the graft modified polysaccharide polymers have new properties that to be tailored by the chemistry of the grafted polymer. For example, responsive polymers can control the drug release of polysaccharide NPs [53]. In the future, the mechanism of action of polysaccharide polymers should be further studied, which might pave the way for more rational design of polysaccharide-based nanoparticle adjuvants [157].

Overall, we have reviewed the natural polysaccharides-based drug delivery systems for the treatment of diabetes. It can be concluded that polysaccharide NPs have proven to be very important future biological nanocarriers. However, still, most of the research for polysaccharide-based nanocarriers was confined to the preclinical setting, and these polysaccharide NPs with potential clinical value still need to be further studied. All these accumulated evidences about the superior biological and physiochemical aspects of polysaccharides are convincing that they could be used as promising biomaterials in future.

## Figures and Tables

**Table 1:** Polysaccharide-based nanocarriers for the entrapment of drugs and used against diabetes.

Polysaccharide	Nanocarriers	Drug	Drug Loading (%)	Encapsulation Efficiency (%)	Particles Size (nm)	Zeta Potential (mV)	Testing Method	Ref
ALG	NPs	INS	10.8	49.6	201.8	−35.7	SC	[107]
ALG	NPs	INS	11.7~38.9	/	40~150	−88~−21.2	In vitro	[108]
ALG	NPs	INS	3.2~3.5	77.2~79.2	202~279.5	−34.3~+14.6	PO	[109]
ALG	NPs	INS	/	22,85	78,91	−27.3, −17.1	PO	[110]
ALG	Emulsions	Metformin	3.1	78	60~150	+27.3	PO	[95]
ALG/CS	NPs	Metformin	/	10.0~21.6	701~989	−29.8~+5.5	PO	[77]
ALG/CS	NPs	Berberine	8.6	85.7	202.2	−14.8	PO	[111]
ALG/CS	NPs	Liraglutide	54.2	92.5	100	/	PO	[112]
ALG/CS	Emulsions	INS	/	47.3	488	−62.25	PO	[96]
ALG/CS	NPs	INS	7.5~13.9	78.9~83.3	797~4895	/	In vitro	[113]
ALG/CS	NPs	INS	/	85	100~200	+9.62~+16.42	PO	[114]
ALG/CS	NPs	Quercetin	59	95	91.58	−24.63	PO	[81]
ALG/CS	NPs	INS	17.25	97	90~110	+38.5	PO	[82]
ALG/CS	NPs	INS	10.7, 4.9	81.5, 55.2	260.1, 224.4	−55.7, +13.7	PO	[115]
ALG/CS/MD	Micelles	Curcumin	3.3~5.0	/	/	/	PO	[88]
ALG/DS	NPs	INS	10.1	72.4	313.2	−30.6	In vitro	[80]
ALG/TMC	NPs	INS	5.4	93.2	150.8	/	In vitro	[116]
ALG/KC	Hydrogel	IASP	/	19~26	2500~2700	/	In vitro	[101]
Cellulose	Hydrogel	NCs	/	/	/	/	Cover the wound	[105]
CMCD/CMC	Hydrogel	INS	30.0~31.9	/	/	/	PO	[117]
CS	NPs	INS	27.5	91.0	46.2	+9.4	PO	[118]
CS	Liposomes	INS	/	85.75	439	+60.5	PO	[119]
CS	Liposomes	Curcumin	/	/	247.6	+61.5	In vitro	[120]
CS	NPs	INS	/	73.0~85.4	204~303.9	+14.9~+32.1	PO	[121]
CS	NPs	INS	/	49.4~60.9	215~255	+20.7~+30.1	PO	[122]
CS	NPs	INS	47.0	52.5	551.7	+25.7	PO	[123]
CS	NPs	INS	/	93.1	91.3~220.2	+14.4	In vitro	[124]
CS	NPs	INS	88.4~98.7	88.4~98.7	86~257	+22.1~+26.1	SC	[125]
CS	Liposomes	FeSO_4_	/	72.8~82.7	209~304	+27.8~+33.1	In vitro	[126]
CS	NPs	INS	13.4~17.2	79.3~82.5	39.2~50.6	+27.8~+49.6	PO	[127]
CS	NPs	INS	35.8~33.0	72.4~78.2	217.6~237.0	+8.5~+15.6	PO	[128]
CS	Liposomes	INS	10.7	/	100~150	−40~+40	PO	[76]
CS	Hydrogel	BSA	9.85~9.91	98.63~99.07	/	/	In vitro	[129]
CS	NPs	FA	/	50	242	+32	In vitro	[130]
CS	NPs	INS	18~19	68~74	218~250	+25~+27	In vitro	[131]
CS	Emulsions	INS	/	/	8.7~141.9	+1.2~+33.9	PO	[132]
CS	NPs	INS	/	85	200~550	+2.83~+24.69	PO	[133]
CS	NPs	INS	6.83	41	252.4	+5.99	PO	[134]
CS	NPs	Exendin-4	14.7	60.9	260~1049	+3.6~+33.7	PO	[135]
CS/fucoidan	Capsules	INS	8.6	56.4	256.7	+26.5	PO	[136]
CS/Pectin	NPs	Metformin	/	60~92	581.8	+41.8	PO	[42]
CS/Pectin	Nanogel	TM	/	27.4	175.2	−10.9	In vitro	[137]
Starch	NPs	Plant extract	/	/	19.8	/	In vitro	[138]
Pectin	NPs	Metformin	/	68	482.7	+38.9	PO	[84]
HA	NPs	/	/	/	221.0	−25.7	IV	[139]
HA	NPs	CD44 siRNAs	/	/	223.1	−25.6	IP	[140]
HA/CS	NPs	INS	8.3	95	95~200	−40~−50	JI	[141]
Xanthan	Micelles	Glibenclamide	/	98.8	652.8	−27.6	PO	[87]
Gelatin	NPs	INS	/	11.7~49.7	50~250	/	In vitro	[142]
Dextran	Polymersome	INS	2.2~27.8	44.2~92.5	123.5~342.3	/	PO	[143]
Dextran	NPs	INS	/	48.68	48~74	/	In vitro	[144]
CS/GA	NPs	Glycyrrhizin	/	99.84	181.4	+31.4	PO	[145]
CMCS	NPs	INS	9.8	67	100~300	/	PO	[146]
Pullulan	Hydrogel	INS	1.9~12.2	/	/	/	In vitro	[102]
Pullulan	Hydrogel	acarbose	/	63	/	/	In vitro	[147]
TMC	Micelles	INS	12.0~14.2	82.2~90.3	106.8~149.8	−19.1~+25.4	PO	[148]
CS/HPMCP	NPs	INS	17.6~48.4	87.8~90.9	168~239	−26.7~+19.5	PO	[85]
CS/β-CD	Hydrogel	INS	/	/	/	/	PO	[103]
CMCD-g-CS	NPs	INS	0.13	57.0	144~297	/	PO	[2]
NTC	NPs	INS	7.8	47.0	247.6	+45.2	PO	[149]
NSC	Hydrogel	INS	38	76	/	/	PO	[150]
β-CD	Nanogel	INS/pCMV-Ins	8.4	84.4	164~240	+23~+46.3	In vitro	[151]
SCG	NPs	INS	2.5~3.5	53.9~70.2	60~200	−2.0~−6.5	PO	[152]
Salecan	NPs	INS	/	30.3~60.8	/	/	PO	[104]

Abbreviations: CMCD/CMC: Carboxymethyl β-cyclodextrin grafted carboxymethyl chitosan, BSA: bovine serum albumin, CMCD-g-CS: carboxymethyl-β-cyclodextrin-grafted chitosan, CMCS: carboxymethyl chitosan, DS: dextran sulfate, FA: Ferulic acid, KC: κ-carrageenan, GA: gum Arabic, IASP: Insulin aspart, SCG: short-chain glucan, FD: fucoidan, MD: maltodextrin, TM: Timolol maleate, TMC: Trimethyl chitosan, HPMCP: hydroxy-propyl methylcellulose phthalate, NTC: N-trimethyl chitosan chloride, NSC: N-succinyl chitosan, NCs: Nanobiocomposites, SC: Subcutaneous injection, IV: intravenous injection, JI: Jejunal instillations, PO: per os, INS: Insulin.

## Data Availability

All the data can be available in Reference.
